# Supplementary material for: GABAergic neurons in the rostromedial tegmental nucleus are essential for rapid eye movement sleep suppression
Source: Nat Commun. 2022 Dec 7;13:7552. doi: 10.1038/s41467-022-35299-x (PMC9729601; doi:10.1038/s41467-022-35299-x)
Supplement: Supplementary file 1 — Supplementary Information [file 41467_2022_35299_MOESM1_ESM.pdf]

Supplementary figures

GABAergic neurons in the rostromedial tegmental nucleus are essential for rapid eye movement sleep suppression

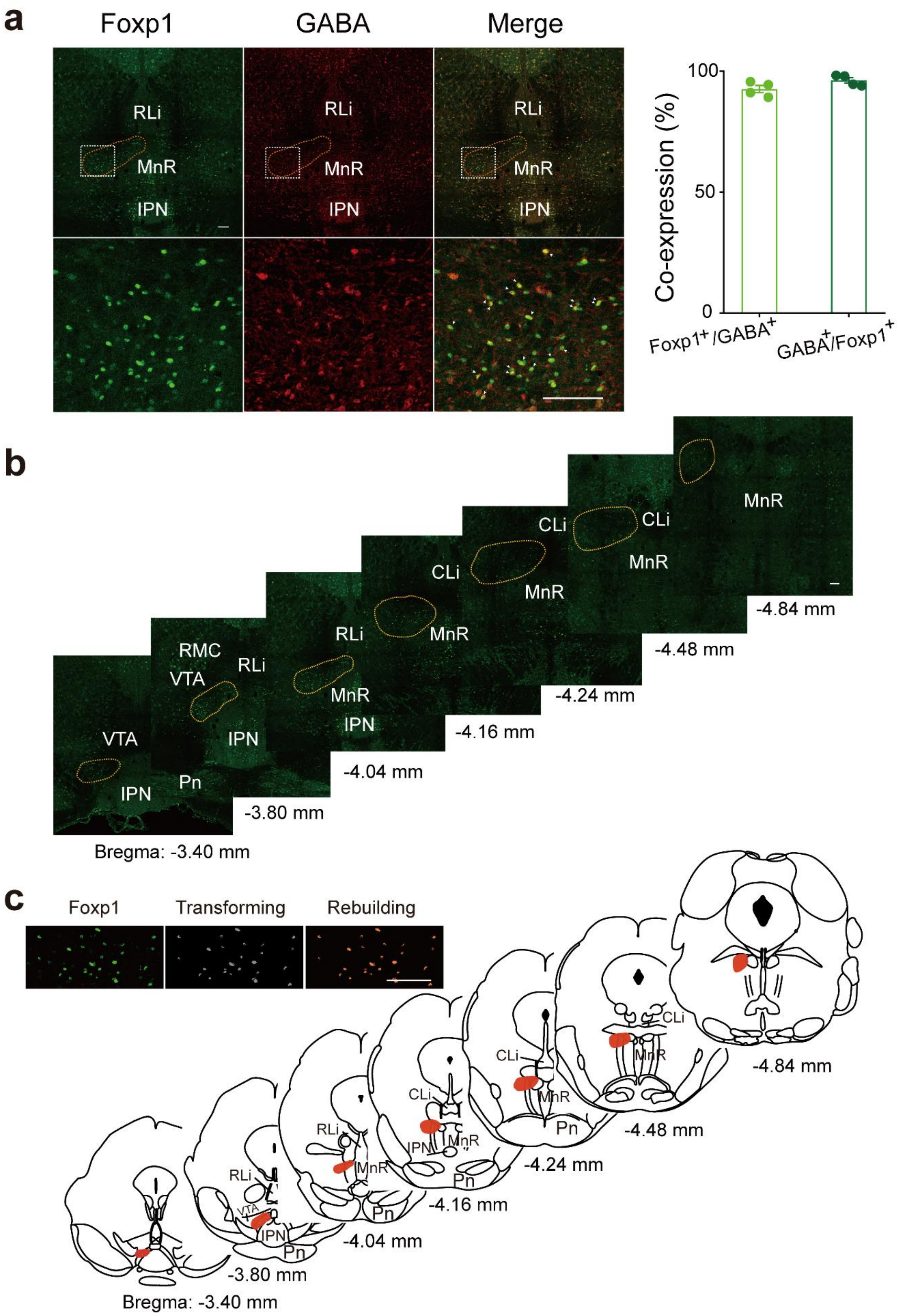
a
Foxp1
GABA
Merge
RLi
MnR
IPN
Co-expression (%)
100
50
0
Foxp1+/GABA+
GABA+/Foxp1+
b
Bregma: -3.40 mm
-3.80 mm
-4.04 mm
-4.16 mm
-4.24 mm
-4.48 mm
-4.84 mm
VTA
RMC
Pn
CLi
c
Foxp1
Transforming
Rebuilding

Fig. S1 RMTg location defined by Foxp1 immunostaining on the mouse atlas. a Left panel, immunostaining of a typical brain section stained against Foxp1 (green) and GABA (red), and co-localized Foxp1 and GABA staining (yellow, the cells pointed to with white arrows). Top, the red circle depicts the RMTg location. Bottom, higher magnification image of the white box in the top pictures. Right panel, quantification of neurons that co-expressed Foxp1 and GABA. n = 4 mice. **b** Foxp1 expression spreads from bregma: −3.40 mm to bregma: −4.84 mm. Dashed circle, the RMTg location defined by Foxp1 expression. **c** Top, transforming and rebuilding of the immunostaining of Foxp1. After Foxp1 was immunostained with green fluorescence, the pictures were captured by a confocal microscope. The images were processed in ImageJ. The RGB images were converted into 8-bit pictures, where the boundary of the RMTg was defined clearly from the adjacent region above a certain threshold in black-and-white images. Then the automatic wand tool was applied, and representative cell bodies were colored and matched to the mouse brain atlas accordingly. Bottom, location of the RMTg defined by Foxp1 staining on the mouse atlas. Scale bar, 50 μm in a−c. Abbreviations: RLi, rostral linear nucleus of the raphe; CLi, caudal linear nucleus of the raphe; RMC, red nucleus, magnocellular part; IPN, interpeduncular nucleus; Pn, pontine reticular nucleus; VTA, ventral tegmental area; MnR, median raphe nucleus. Source data are provided as a Source Data file.

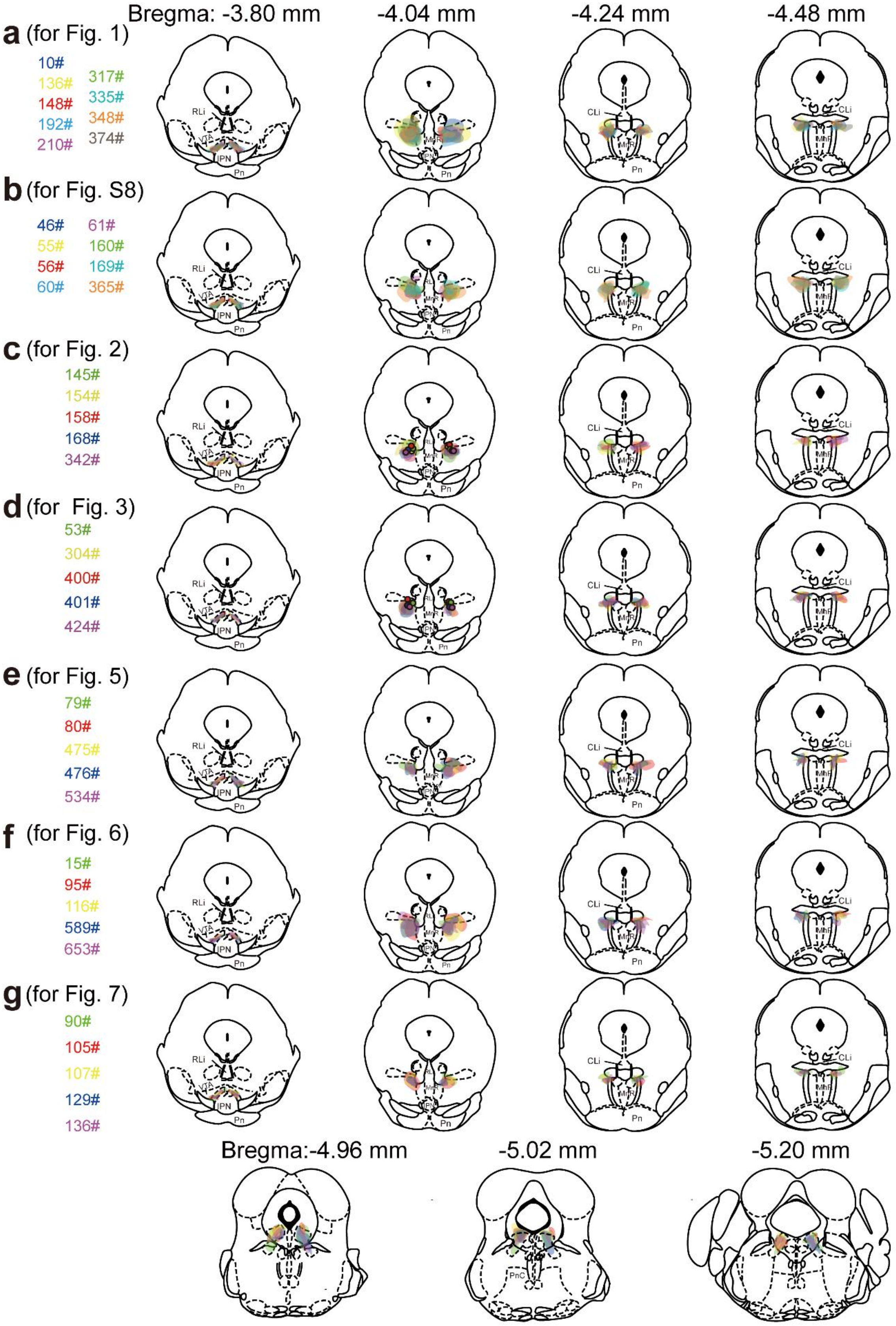
Bregma: -3.80 mm
-4.04 mm
-4.24 mm
-4.48 mm
a (for Fig. 1)
10#
136#
148#
192#
210#
317#
335#
348#
374#
b (for Fig. S8)
46#
55#
56#
60#
61#
160#
169#
365#
c (for Fig. 2)
145#
154#
158#
168#
342#
d (for Fig. 3)
53#
304#
400#
401#
424#
e (for Fig. 5)
79#
80#
475#
476#
534#
f (for Fig. 6)
15#
95#
116#
589#
653#
g (for Fig. 7)
90#
105#
107#
129#
136#
RLi
VTA
IPN
Pn
MnR
CLi
Bregma:-4.96 mm
-5.02 mm
-5.20 mm
PnC

Fig. S2 AAVs-microinjected area of each tested mouse in different behavioral experiments. a–f In the experiments observing sleep–wake states by manipulating RMTg GABAergic neurons (**a–d**) or the circuits of $RMTg^{GABA}$-LDT (**e, g**) and $RMTg^{GABA}$-LH (**f**), the coronal sections at four brain levels from bregma −3.80 mm to bregma −4.48 mm show the superimposed area of the RMTg GABAergic neurons expressing AAVs encoding hM3Dq (**a**), hM4Di (**b**), ChR2 (**c, e, f, g top**), and Arch (**d**) and the superimposed area of the LDT neurons expressing shVglut2 at three brain levels from bregma −4.96 mm to bregma −5.20 mm (**g**, bottom) in each mouse.

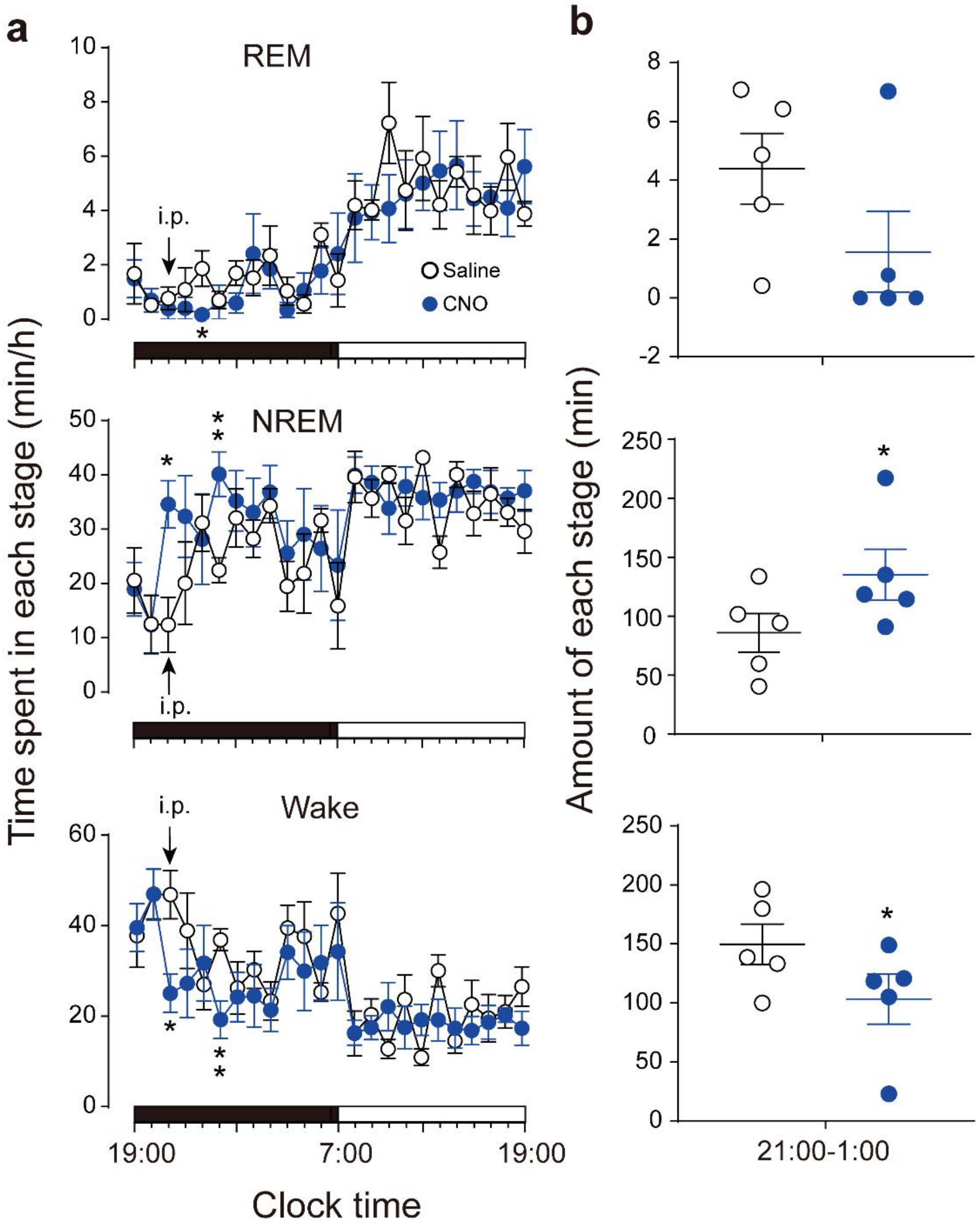

Fig. S3 Chemogenetic activation of RMTg GABAergic neurons during the active period promoted NREM sleep. a, b Sleep–wake quantities following administration of saline or clozapine-N-oxide (CNO), including the average hourly (**a**) and total sleep–wake amounts of REM sleep ($T_4 = 1.696$, $p = 0.1651$), NREM sleep ($T_4 = 3.561$, $p = 0.0236$), and wakefulness ($T_4 = 3.478$, $p = 0.0254$) during the 4-h post-injection period (21:00–1:00) (**b**). *$p < 0.05$, **$p < 0.01$. Statistics using two-way repeated ANOVA followed by multiple paired *t* test in **a** and using paired *t* test in **b**. Data represent mean ± SEM. $n = 5$. Source data are provided as a Source Data file.

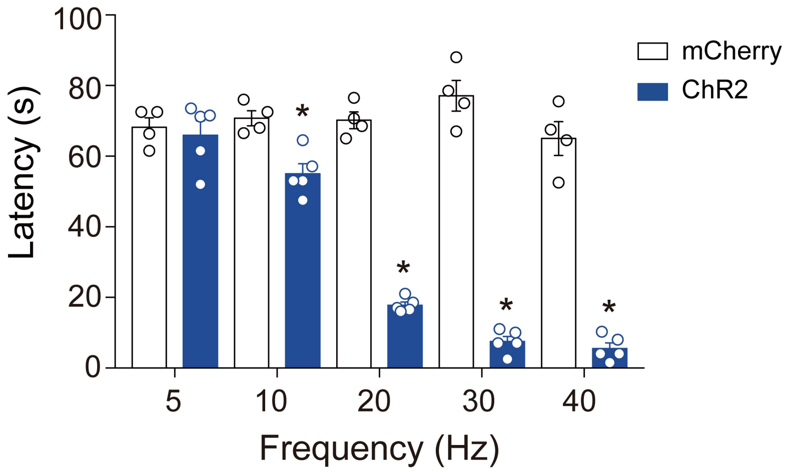

Fig. S4 Latencies of transitions from REM sleep after photostimulation of RMTg GABAergic neurons at different frequencies. Laser stimulation was applied during REM sleep lasting no less than 16 s before laser on. The duration of light illumination was 120 s with laser pulse increased from 5 Hz to 40 Hz. The light simulation with each frequency was repeated at least eight trials for each animal. The interval between two trials of light stimulation was no less than 10 min. The latencies of REM sleep transitions to wakefulness or NREM sleep among the first five to eight trials for each animal were calculated at different stimulation frequencies. With the frequency of blue light illumination increased from 5 Hz to 40 Hz, the REM sleep conversion latency gradually decreased and became more remarkable compared with the mCherry group. p = 0.0159, Wilcoxon signed rank test. Data represent mean ± SEM. ChR2: n = 5, mCherry: n = 4. Source data are provided as a Source Data file.

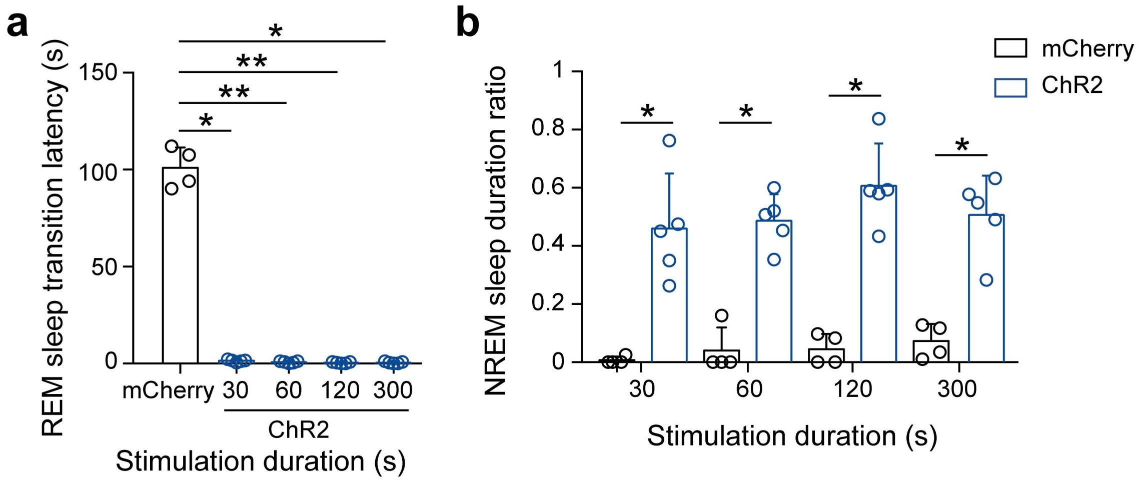

Fig. S5 REM sleep transition latency and NREM sleep duration ratio across different durations of laser stimulations. a REM sleep transition latency across different durations of laser stimulation in VGAT-Cre mice (laser onset during REM sleep). **b** NREM sleep duration ratio, calculated as follows: NREM sleep duration during the time of laser stimulation divided by time of laser stimulation in VGAT-Cre mice (laser onset during wakefulness). Mann–Whitney test between the ChR2 and mCherry groups. Wilcoxon signed rank test within the ChR2 group. *$p < 0.05$, **$p < 0.01$. Data represent mean ± SEM. ChR2: $n = 5$, mCherry: $n = 4$. Source data are provided as a Source Data file.

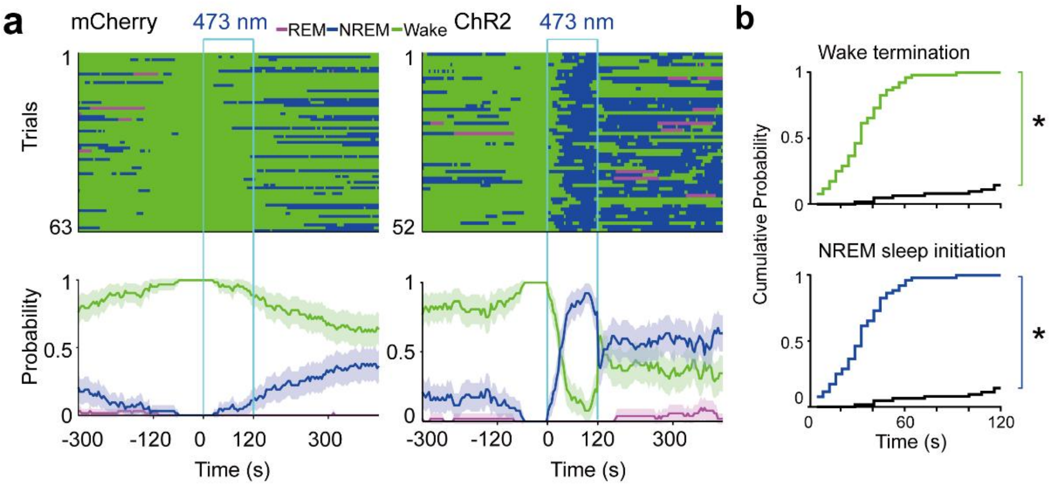

Fig. S6 Optogenetic activation of RMTg GABAergic neurons promoted wakefulness to NREM sleep. a Sleep–wake state changes (top) and probability of brain state transitions (bottom) after photostimulation during an awake state lasting no less than 60 s before laser on in all trials from mice in the mCherry (left) and ChR2 (right) groups, respectively. Shading indicates 95% confidence intervals. **b** During 120 s laser stimulation, cumulative probability for wake termination and NREM sleep initiation. Colorful stairs, ChR2 group. Black stairs, mCherry group. *$p < 0.05$ vs control group, Kolmogorov–Smirnov test. Data represent mean ± SEM. ChR2: $n = 5$, mCherry: $n = 4$. Source data are provided as a Source Data file.

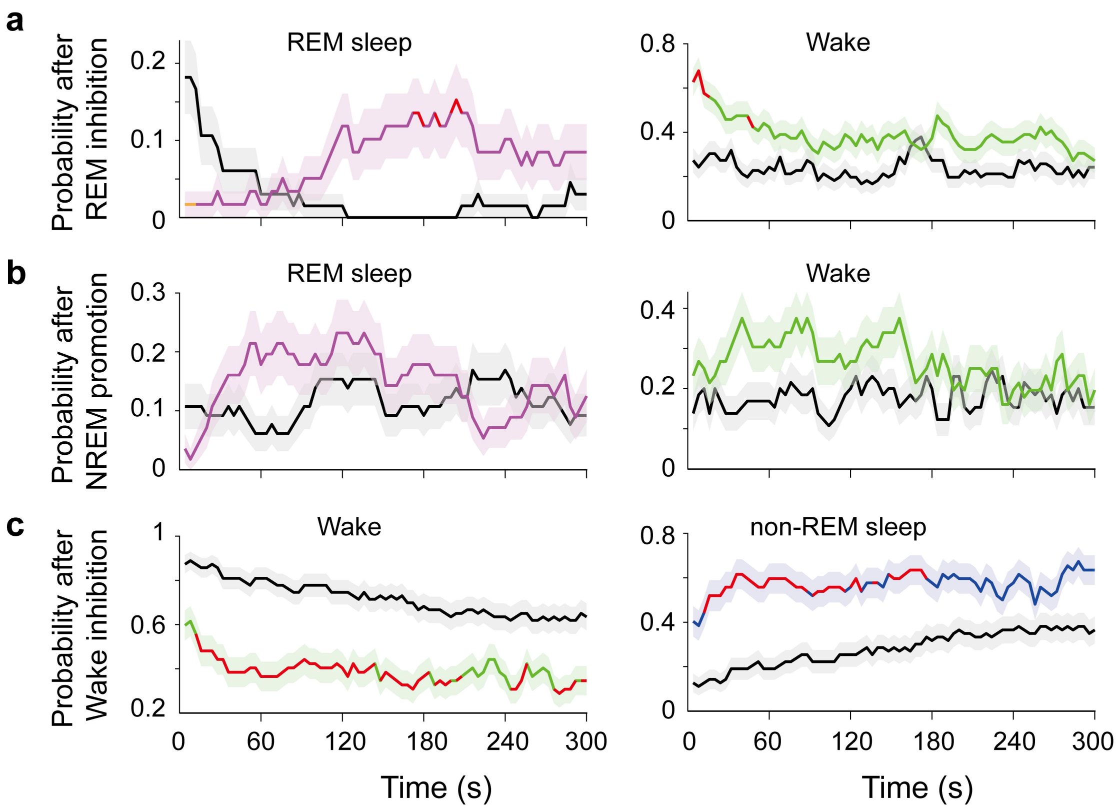

Fig. S7 Rebound of each vigilance state after the end of optogenetic activation of RMTg GABAergic neurons. During 300 s after the end of 120-s laser stimulation of RMTg GABAergic neurons, the probability of REM sleep (left) and wakefulness (right) after REM sleep inhibition in Fig. 2e (**a**) and after NREM sleep promotion in Fig. 2h (**b**) and probability of wakefulness (left) and NREM sleep (right) after wake inhibition in Fig. S5a (**c**). Black, control group. The colorful, experimental group (green, wakefulness; magenta, REM sleep; blue, NREM sleep). Red, values with significant differences (Two-way ANOVA followed by a post hoc Sidak test). Shading, SEM. Source data are provided as a Source Data file.

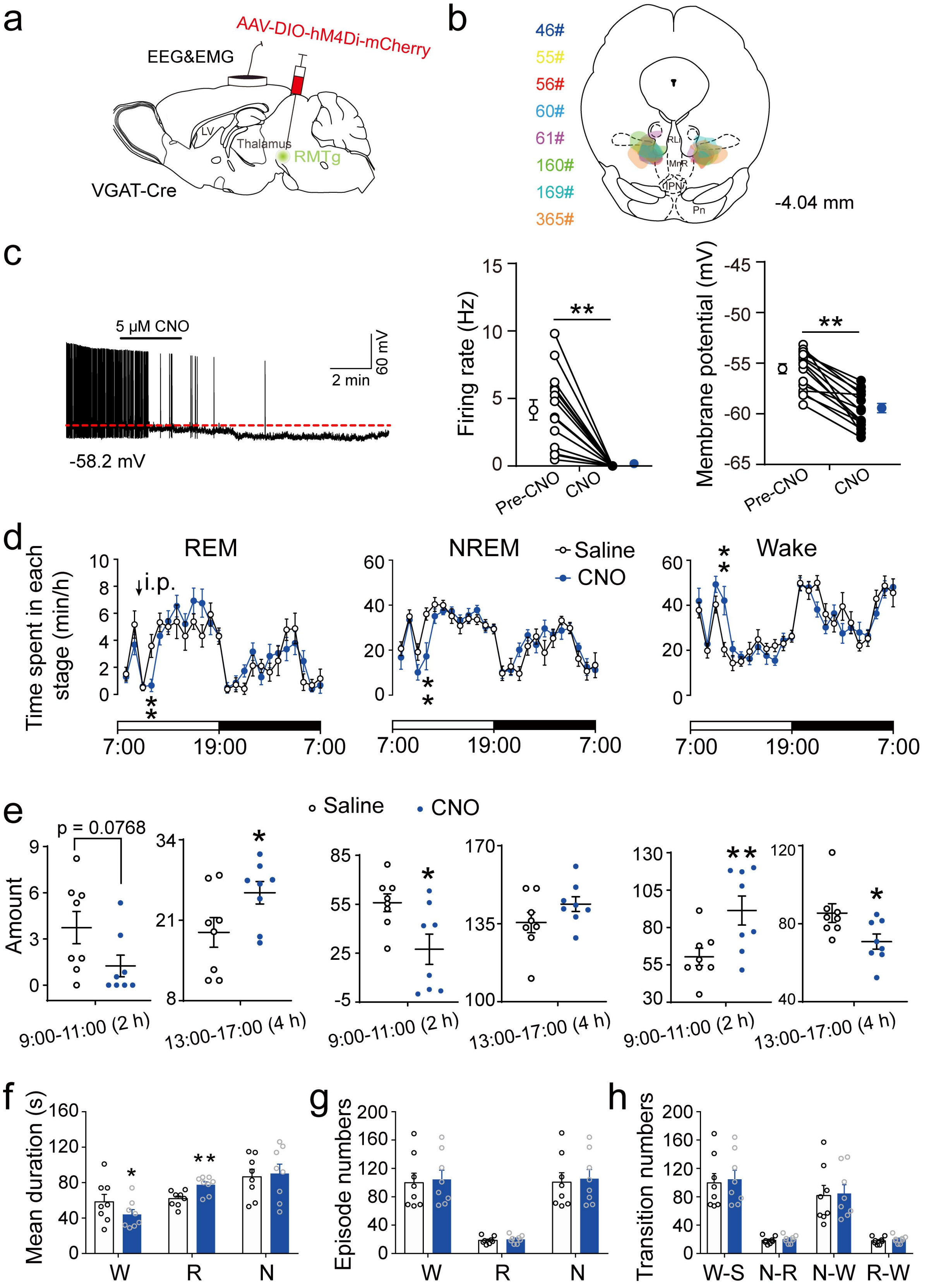

Fig. S8 Chemogenetic inactivation of RMTg GABAergic neurons promoted REM sleep and wakefulness. a Schematic illustration of virus injection. Adeno-associated viruses

(AAVs) encoding hM4Di fused to an enhanced red fluorescent protein (mCherry) were introduced into the bilateral RMTg in VGAT-Cre mice. **b** The coronal section shows the superimposed virus-injected area in eight mice. **c** A typical trace showed that application of clozapine-N-oxide (CNO) inhibited spontaneous firing and decreased the membrane potential of an hM4Di-expressing RMTg GABAergic neuron. The average firing rates (T_{14} = 5.385, p < 0.0001) and membrane potential (T_{14} = 7.997, p < 0.0001) of RMTg hM4Di-expressing neurons (N = 15 cells in 5 mice) were significantly decreased by CNO application. The results from each cell are shown on the scatter plot. **d, e** After administration of saline or CNO, the hourly average amount of each stage [two-way repeated measures ANOVA followed by a *post hoc* Sidak test: $F_{1,14}$ = 5.338, p = 0.0366 (REM); $F_{1,14}$ = 6.789, p = 0.0208 (NREM); $F_{1,14}$ = 7.388, p = 0.0167 (wake) during 9:00–11:00; $F_{1,14}$ = 4.314, p = 0.0567 (REM); $F_{1,14}$ = 3.828, p = 0.0706 (NREM); $F_{1,14}$ = 7.089, p = 0.0186 (wake) during 13:00–17:00] (**d**) and total amount of each stage during the 2-h (9:00–11:00, left) and 4-h (13:00–17:00, right) post-injection period [T_7 = 3.169, p = 0.0157 (13:00–17:00: REM sleep); T_7 = 3.361, p = 0.0121 (9:00–11:00: NREM sleep); T_7 = 3.531, p = 0.0096 (9:00–11:00: wake); T_7 = 3.366, p = 0.012 (13:00–17:00: wake) (**e**). **f–h** Sleep–wake architecture during the 4-h post-injection period (13:00–17:00) after saline or CNO administration, including mean duration (W: T_7 = 3.06, p = 0.0183; R: T_7 = 4.831, p = 0.0019) (**f**) and episode number (**g**) in different sleep–wake states and conversions between W (wakefulness), N (NREM sleep), and R (REM sleep) (**h**). *p < 0.05, **p < 0.01. Statistics obtained by paired t tests are shown in e–h. Data represent mean ± SEM. n = 8. Source data are provided as a Source Data file.

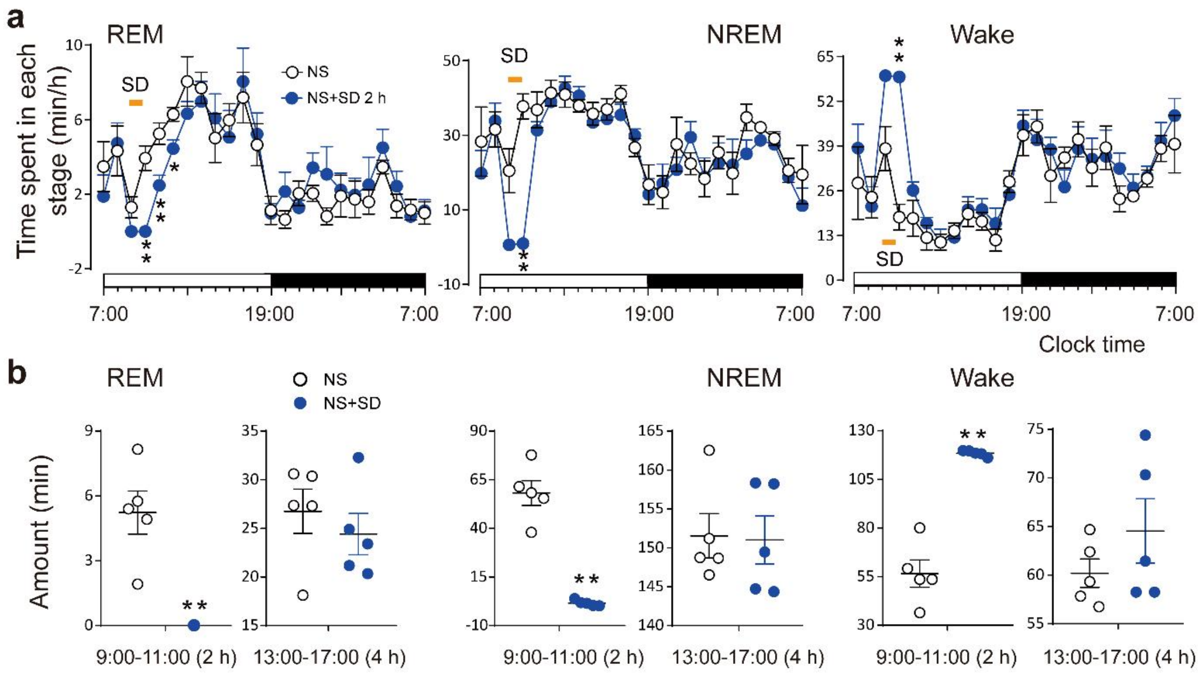

Fig. S9 Sleep deprivation for 2 h did not cause any rebound of REM and NREM sleep in VGAT-Cre mice. a The hourly average amount of each stage [two-way repeated ANOVA followed by a post hoc Sidak test: $F_{1,8} = 1119.1$, $p < 0.01$ (REM); $F_{1,8} = 40.55$, $p < 0.01$ (NREM); $F_{1,8} = 50.35$, $p < 0.01$ (wake) during 9:00–13:00] after saline administration at 9:00 and followed by 2-h sleep deprivation (SD). **b** Total amount of each stage during the 2-h SD period (9:00–11:00, left, REM sleep: $T_4 = 5.239$, $p = 0.0063$; NREM sleep: $T_4 = 8.559$, $p = 0.0010$; Wake: $T_4 = 8.618$, $p = 0.0010$) and 4-h period after saline injection (13:00–17:00, right). Statistics by paired t test. $^*p < 0.05$, $^{**}p < 0.01$ vs. saline not SD group. Data represent mean ± SEM. $n = 5$. Source data are provided as a Source Data file.

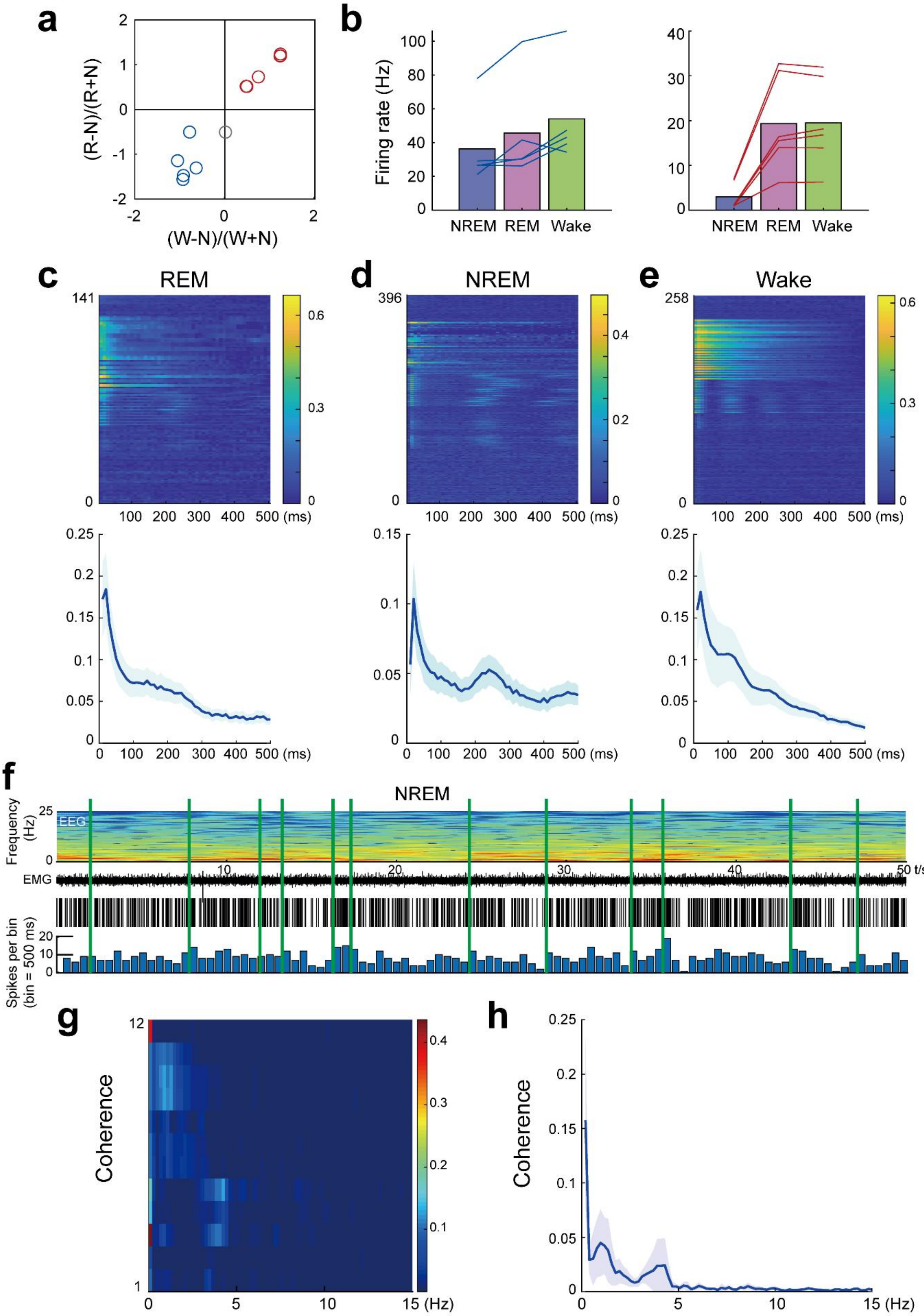

a
(R-N)/(R+N)
(W-N)/(W+N)
b
Firing rate (Hz)
NREM REM Wake
c
REM
d
NREM
e
Wake
(ms)
f
NREM
Frequency (Hz)
EEG
EMG
50 t/s
Spikes per bin (bin = 500 ms)
g
Coherence
15 (Hz)
h
Coherence
15 (Hz)

Fig. S10 RMTg GABAergic units firing characteristics across distinct sleep–wake states. **a** Firing rate modulation of 12 identified units from 3 mice (z-scored). W: wake, R: REM sleep, N: NREM sleep ($p < 0.05$, Wilcoxon rank-sum test). **b** Firing rates of significant NREM-active (blue) and NREM-inactive (red) neurons during different brain states. Each line shows the firing rates of one unit; bar, average firing rates of the units. **c**–**e** Autocorrelograms for firing patterns of RMTg GABAergic units in each brain state. Each row represents one trial of REM sleep (**c**), NREM sleep (**d**), and wakefulness (**e**) (top). Average of autocorrelation of each brain state (bottom). Shading, 95% confidence intervals. **f** Representative firing rates of a RMTg GABAergic neuron together with EEG spectrogram and EMG amplitude. Green lines indicate time point of high delta oscillation (0.65–4 Hz). **g** Spike-EEG spectrogram coherence of each identified unit. **h** Average coherence in g. Shading, 95% confidence intervals. Source data are provided as a Source Data file.

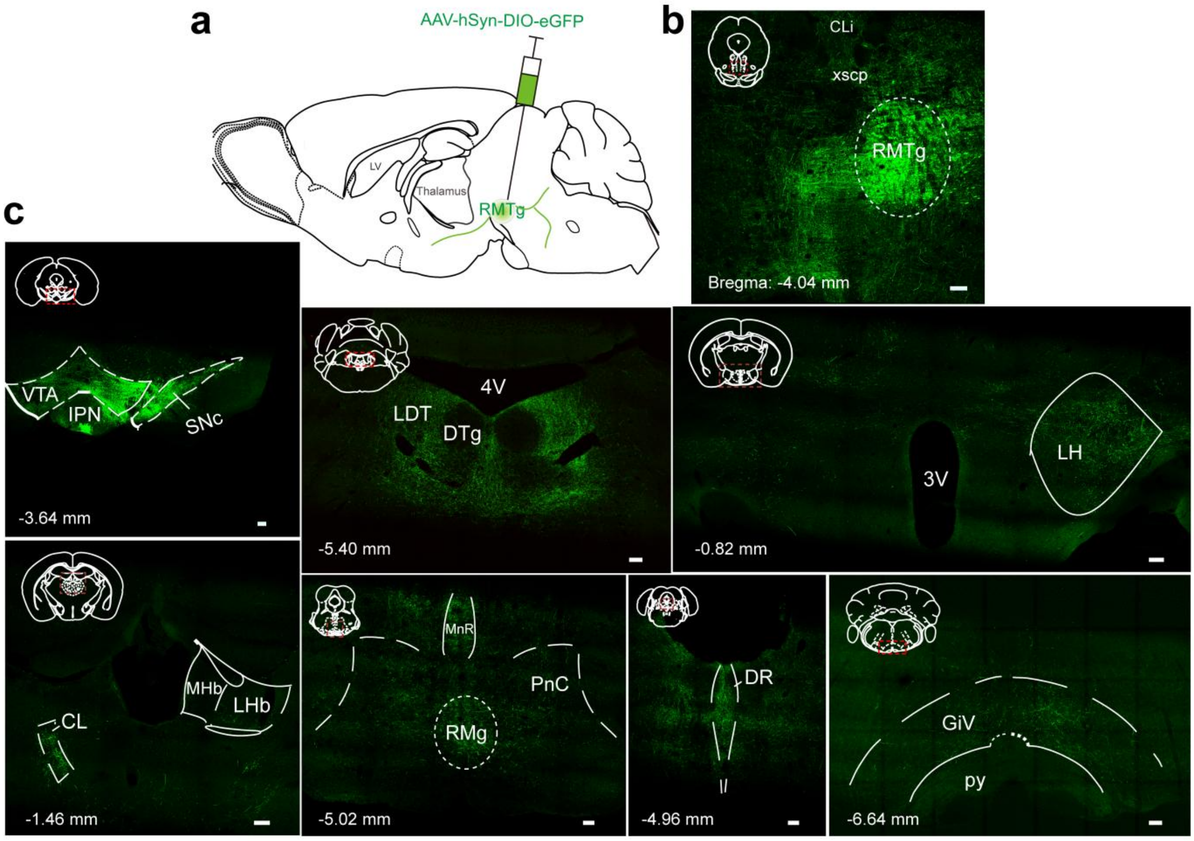

Fig. S11 Antegrade tracing of RMTg GABAergic neurons. a Schematic illustration of virus injection. Cre-dependent adeno-associated viruses (AAVs) encoding eGFP reporter were injected into the unilateral RMTg in VGAT-Cre mice. **b** A representative fluorescent image showing the unilateral AAV injection site on the RMTg. Dashed circular line depicting the RMTg. **c** Targets innervated by RMTg GABAergic axon terminals. Abbreviations: CLi, caudal linear nucleus of the raphe; xscp, decussation of the superior cerebellar peduncle; VTA, ventral tegmental area; IPN, interpeduncular nucleus; substantia nigra compacta (SNc); LDT, laterodorsal tegmental nucleus; DTg, dorsal tegmental nucleus; 4V, 4th ventricle; LH, lateral hypothalamus; 3V, 3rd ventricle; MHb, median habenula, LHb, lateral habenula; CL, centrolateral thalamic nucleus; RMg, raphe magnus nucleus; MnR, median raphe nucleus; PnC, pontine reticular nucleus, caudal part; DR, dorsal raphe; GiV, gigantocellular reticular nucleus, ventral part; py, pyramidal tract. Scale bar, 100 μm.

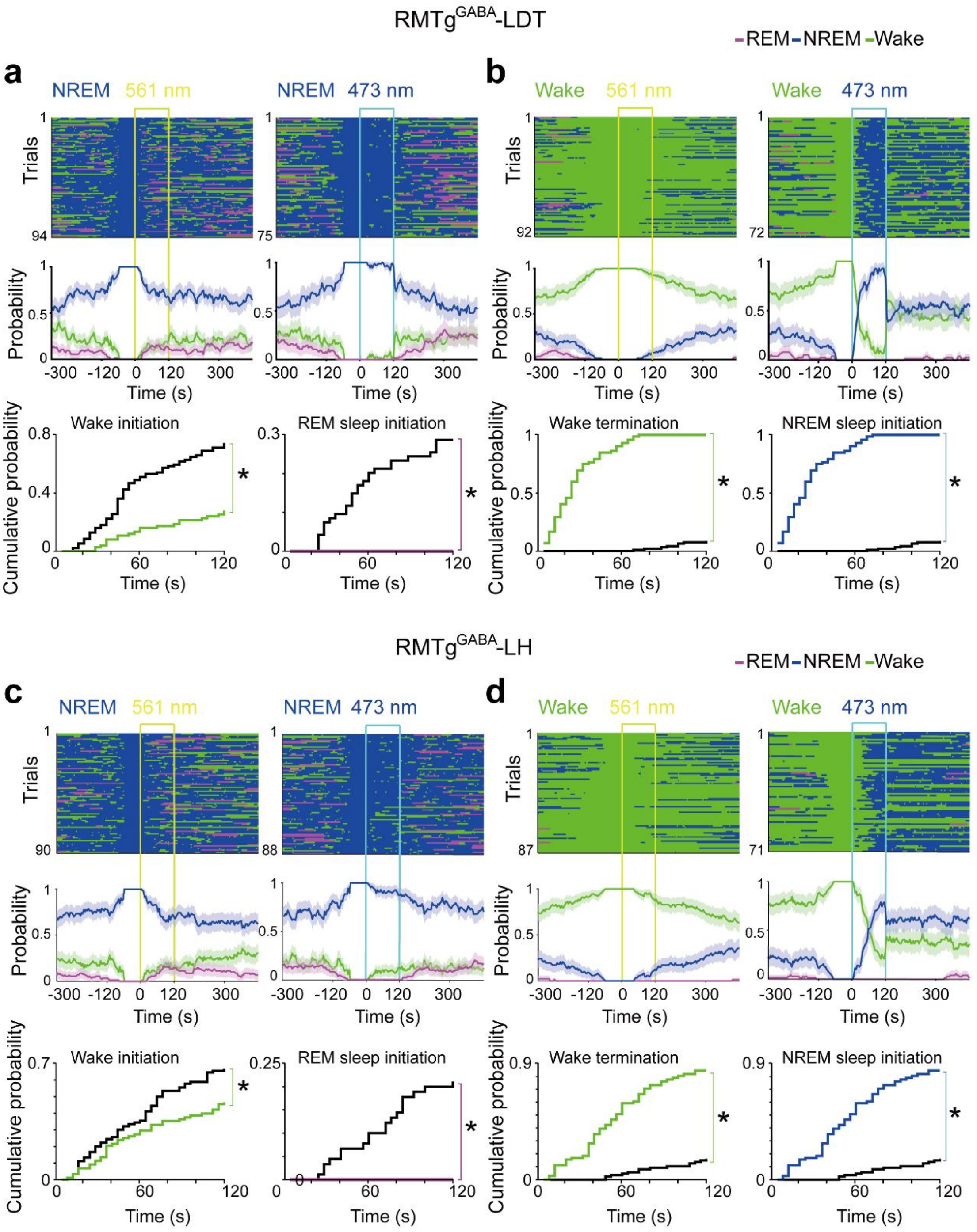

Fig. S12 Optogenetic activation of the RMTg^{GABA}-LDT or RMTg^{GABA}-LH promoted NREM sleep. a–d During 120 s laser stimulation, sleep–wake state changes (top), probability (middle), and cumulative probability (bottom) of brain state transitions after photostimulation of RMTg GABAergic axon endings in the LDT (**a, b**) or LH (**c, d**) during NREM sleep (**a, c**) or the awake state (**b, d**) lasting no less than 60 s before laser on in all

trials from mice in the $RMTg^{GABA}$-LDT or $RMTg^{GABA}$-LH circuit group with 561 nm laser stimulation as the control (left) and 473 nm laser stimulation (right), respectively. Shading indicates 95% confidence intervals. Yellow and blue line, 561 nm and 473 nm laser stimulation (30 Hz, 10 ms per pulse, 120 s). Colorful stairs, ChR2 group with 473 nm laser illumination. Black stairs, ChR2 group with 561 nm laser illumination as control. $^{*}p < 0.05$ vs control group, Kolmogorov–Smirnov test. Data represent mean ± SEM. $n = 5$. Source data are provided as a Source Data file.

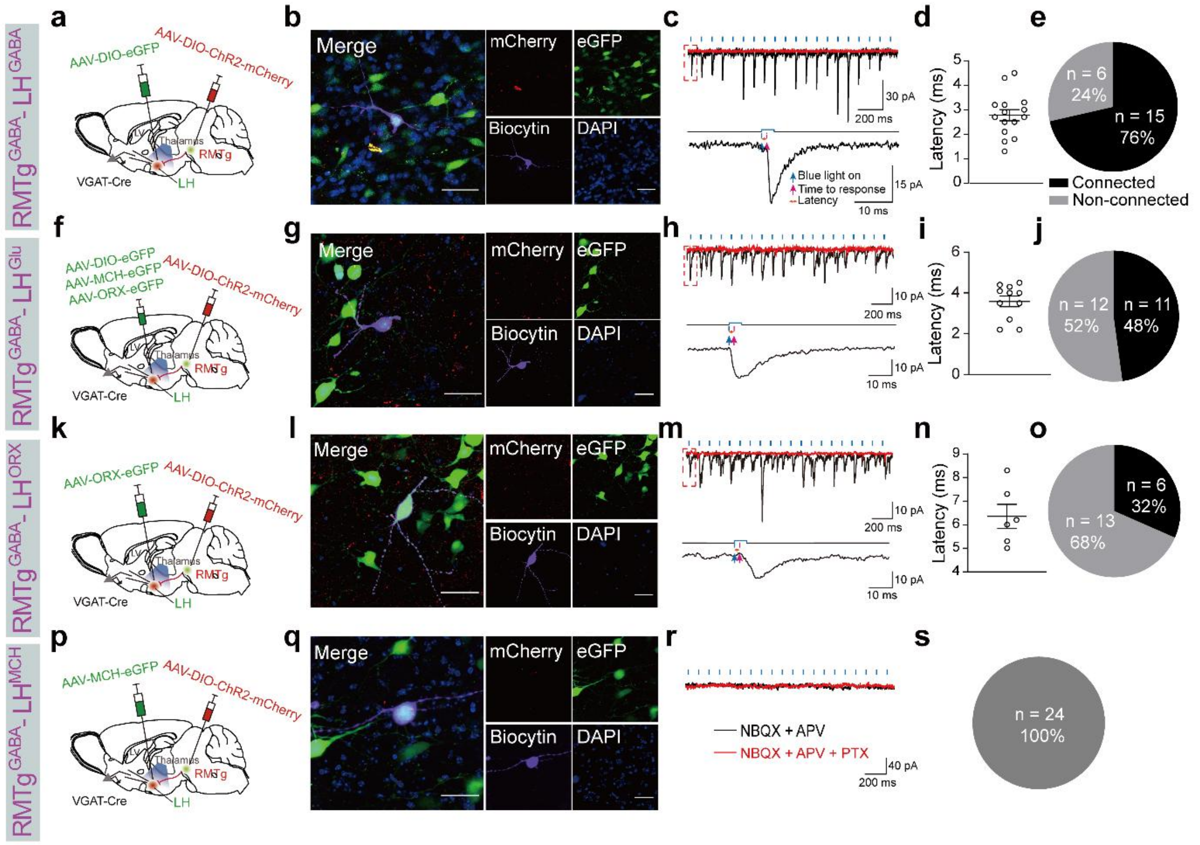

Fig. S13 Direct inhibitory connections between RMTg GABAergic neurons and LH GABAergic and glutamatergic neurons. a, f, k, p Schematic diagrams of the experimental protocols. Cre-dependent adeno-associated viruses (AAVs) encoding ChR2 were introduced into the RMTg, whereas Cre-dependent AAVs encoding eGFP (**a**); three kinds of AAVs encoding eGFP, promoter of orexin neurons (ORX-eGFP), and promoter of melanin-concentrating hormone (MCH) neurons (MCH-eGFP) (**f**); AAVs encoding ORX-eGFP (**k**), and MCH-eGFP (**p**) were injected into the lateral hypothalamus (LH) of VGAT-Cre mice. **b, g, l, q** Representative images showing a recorded biocytin-filled neuron (violet) that is a GABAergic neuron (eGFP: green) (**b**), a glutamatergic neuron with no eGFP expression (**g**), an orexin neuron (ORX-eGFP) (**l**), and MCH neurons (MCH-eGFP) (**q**). Infected RMTg GABAergic terminals in the LH (mCherry: red) were optogenetically activated. Scale bar: 50 μm. **c, h, m, r** Typical traces of postsynaptic currents from an LH GABAergic (**c**), glutamatergic (**h**), orexin (**m**), and MCH (**r**) neuron evoked by blue light stimulation (black

line). The evoked currents were completely abolished by application of picrotoxin (PTX) (**c, h, m**, red line). **d, i, n** Latency of light-evoked postsynaptic currents in LH GABAergic (**d**), glutamatergic (**i**), and orexin (**n**) neurons. Data represent mean ± SEM. **e, j, o, s** Proportion of recorded LH GABAergic (**e**), glutamatergic (**j**), and orexin (**o**) neurons that responded to laser stimulation of ChR2-expressed RMTg GABAergic terminals, while MCH neurons did not respond to blue light stimulation (**r, s**). $N = 21$ cells (**e**), $N = 23$ cells (**j**), and $N = 19$ cells (**o**) from 3 mice, and $N = 24$ cells (**s**) from 4 mice. Source data are provided as a Source Data file.

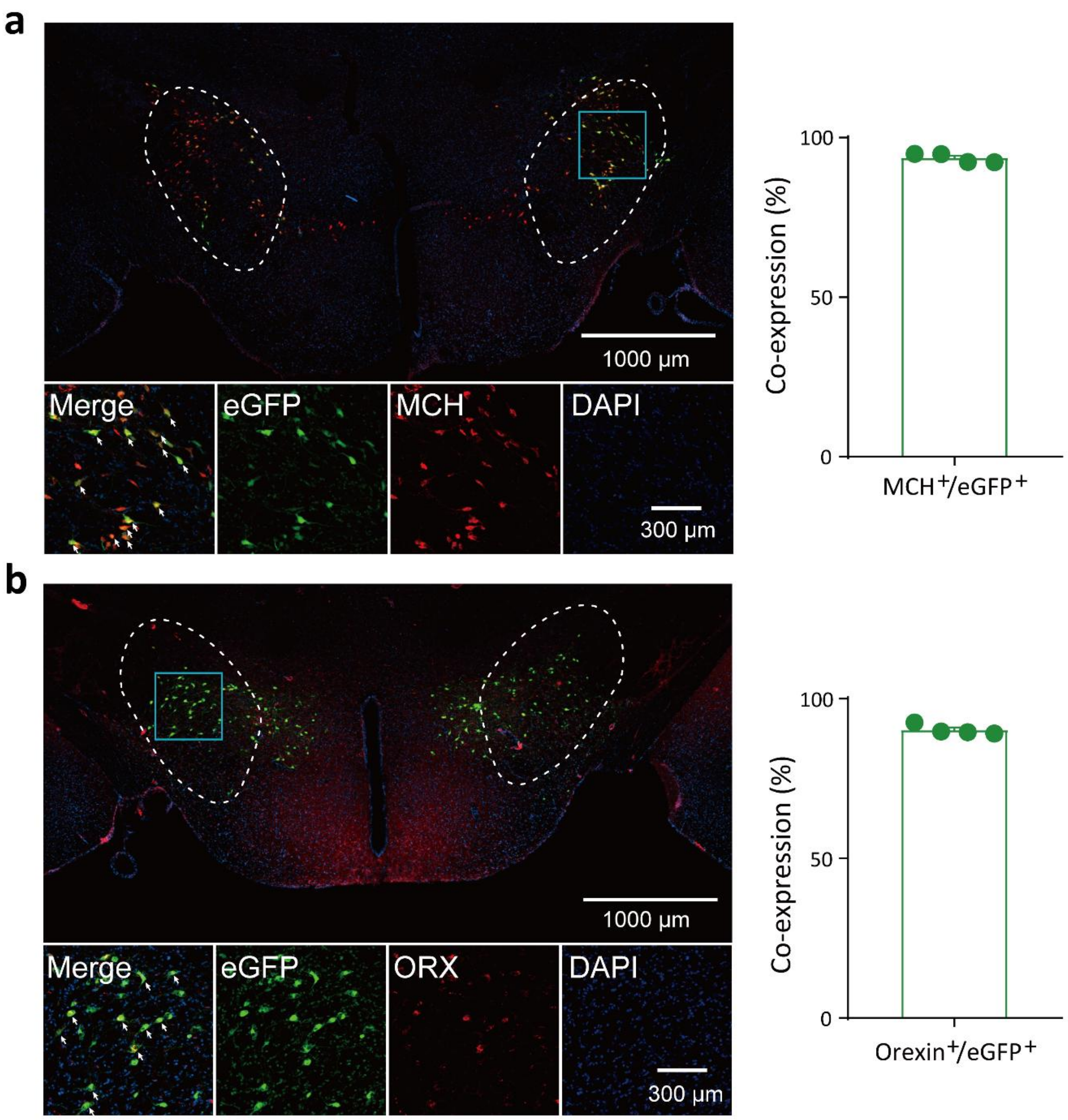

Fig. S14 Co-expression of AAVs encoding specific promotors with several cell markers in the LH. Images showing that green neurons with infection of AAV-MCH-eGFP (**a**) or AAV-orexin-eGFP (**b**) colocalized with MCH and ORX marked by red fluorescence. Merged cells (yellow) are pointed to by white arrows. Top, the white circle depicts the LH location. Bottom, higher magnification image of the blue box in the top pictures. Right column, quantification of AAV-infected green cells that co-expressed specific cell type biomarkers of MCH (**a**) and ORX (**b**). MCH, melanin-concentrating hormone; ORX, orexin; DAPI, 4', 6-diamidino-2-phenylindole. Source data are provided as a Source Data file.
