# Supplementary material for: GABAergic neurons in the rostromedial tegmental nucleus are essential for rapid eye movement sleep suppression
Source: Nat Commun. 2022 Dec 7;13:7552. doi: 10.1038/s41467-022-35299-x (PMC9729601; doi:10.1038/s41467-022-35299-x)
Supplement: Supplementary file 3 — Reporting Summary [file 41467_2022_35299_MOESM3_ESM.pdf]

nature portfolio

Corresponding author(s): Wei-Min Qu, Zhi-Li Huang, Su-Rong Yang

Last updated by author(s): Nov.2, 2022

Reporting Summary

Nature Portfolio wishes to improve the reproducibility of the work that we publish. This form provides structure for consistency and transparency in reporting. For further information on Nature Portfolio policies, see our Editorial Policies and the Editorial Policy Checklist.

Statistics

For all statistical analyses, confirm that the following items are present in the figure legend, table legend, main text, or Methods section.

n/a	Confirmed	
☐	☒	The exact sample size (*n*) for each experimental group/condition, given as a discrete number and unit of measurement
☐	☒	A statement on whether measurements were taken from distinct samples or whether the same sample was measured repeatedly
☐	☒	The statistical test(s) used AND whether they are one- or two-sided<br>*Only common tests should be described solely by name; describe more complex techniques in the Methods section.*
☒	☐	A description of all covariates tested
☐	☒	A description of any assumptions or corrections, such as tests of normality and adjustment for multiple comparisons
☐	☒	A full description of the statistical parameters including central tendency (e.g. means) or other basic estimates (e.g. regression coefficient) AND variation (e.g. standard deviation) or associated estimates of uncertainty (e.g. confidence intervals)
☐	☒	For null hypothesis testing, the test statistic (e.g. *F*, *t*, *r*) with confidence intervals, effect sizes, degrees of freedom and *P* value noted<br>*Give P values as exact values whenever suitable.*
☒	☐	For Bayesian analysis, information on the choice of priors and Markov chain Monte Carlo settings
☒	☐	For hierarchical and complex designs, identification of the appropriate level for tests and full reporting of outcomes
☒	☐	Estimates of effect sizes (e.g. Cohen's *d*, Pearson's *r*), indicating how they were calculated

Our web collection on statistics for biologists contains articles on many of the points above.

Software and code

Policy information about availability of computer code

Data collection	EEG&EMG were recorded using VitalRecorder (Kissei Comtec, Nagano, Japan), in vivo spiking data were recorded with omniplex server of HK Plexon with the help of software SOLIDWORKS 2018.
Data analysis	Data analysis was performed using offline sorter V4, MATLAB R2018b and Prism 7.

For manuscripts utilizing custom algorithms or software that are central to the research but not yet described in published literature, software must be made available to editors and reviewers. We strongly encourage code deposition in a community repository (e.g. GitHub). See the Nature Portfolio guidelines for submitting code & software for further information.

Data

Policy information about availability of data

All manuscripts must include a data availability statement. This statement should provide the following information, where applicable:

- Accession codes, unique identifiers, or web links for publicly available datasets
- A description of any restrictions on data availability
- For clinical datasets or third party data, please ensure that the statement adheres to our policy

Source data are provided with this paper. The codes that support the findings of the present study are available from the corresponding author upon request.

Human research participants

Policy information about studies involving human research participants and Sex and Gender in Research.

Reporting on sex and gender	N/A
Population characteristics	N/A
Recruitment	N/A
Ethics oversight	N/A

Note that full information on the approval of the study protocol must also be provided in the manuscript.

Field-specific reporting

Please select the one below that is the best fit for your research. If you are not sure, read the appropriate sections before making your selection.

☒ Life sciences ☐ Behavioural & social sciences ☐ Ecological, evolutionary & environmental sciences

For a reference copy of the document with all sections, see nature.com/documents/nr-reporting-summary-flat.pdf

Life sciences study design

All studies must disclose on these points even when the disclosure is negative.

Sample size	For chemogenetics, sample size was 8-9 mice. For optogenetics, we used 4-5 mice. There was no sample size calculation to predetermine the sample size. The results showed significant differences compared with the control group. Additionally, the animal ethics requires minimizing the number of animals used. We also referred other articles such as Li et al., Mol Psychiatry 2021, 26(7):2912-2928 and Anaclet et al., Nat Neurosci 2014, 17(9):1217-24.
Data exclusions	Mice with precise AAV injection sites were all included in this study.
Replication	We recorded 2-8 mice each time and repeated 4-6 times in behavioral experiments. For spike and in patch-clamp recording, we repeated 3-5 mice.
Randomization	Mice were randomly assigned to control or experimental group.
Blinding	Investigators were blind to group allocation during data collection and analysis.

Reporting for specific materials, systems and methods

We require information from authors about some types of materials, experimental systems and methods used in many studies. Here, indicate whether each material, system or method listed is relevant to your study. If you are not sure if a list item applies to your research, read the appropriate section before selecting a response.

Materials & experimental systems

n/a	Involved in the study	
☐	☒	Antibodies
☒	☐	Eukaryotic cell lines
☒	☐	Palaeontology and archaeology
☐	☒	Animals and other organisms
☒	☐	Clinical data
☒	☐	Dual use research of concern

Methods

n/a	Involved in the study	
☒	☐	ChIP-seq
☒	☐	Flow cytometry
☒	☐	MRI-based neuroimaging

Antibodies

Antibodies used	Foxp1: Rabbit anti-Foxp1, 1:20,000, ab16645, Abcam, USA;<br>GABA: rabbit anti-GABA, 1:1000; PA5-32241, Invitrogen, USA;<br>Hcrt/orexin: mouse anti-hypocretin-A,1:600, sc-80263, Santa Cruz Biotechnology, USA;<br>MCH: rabbit anti-melanin-concentrating-hormone, 1:1000, M8440, Sigma-Aldrich, USA;<br>Glutamate: rabbit anti-glutamate, 1:1000, G6642, Sigma, USA and Biocytin: 1:1000, S21374, Invitrogen, USA;<br>Donkey anti-rabbit/goat, 1:1000; Jackson ImmunoResearch, USA
Validation	Foxp1 antibodies was validated by Smith et al., Brain structure & function 2019, 224 (1), 219-238. GABA antibodies was validated by Ryu, B et al., Cell reports 2021, 35 (3), 109001. Hcrt/orexin antibodies was validated by Duffet, L et al., Nature methods 2022, 19 (2), 231-241. MCH antibodies was validated by Izawa, S et al., Science (New York, N.Y.) 2019, 365 (6459), 1308-1313. Glutamate antibodies was validated by Storm-Mathisen, J., et al., In: "Excitatory Amino Acids," Roberts, P., et al., (eds.), pp. 101, McMillan (1986). Donkey anti-rabbit/goat was validated by Thau-Zuchman O et al., Front Neurosci 2022, 16: 926023.

Animals and other research organisms

Policy information about studies involving animals; ARRIVE guidelines recommended for reporting animal research, and Sex and Gender in Research

Laboratory animals	Chemogenetic and optogenetic experiments and in vivo electrophysiological recording were performed in male vesicular GABA transporter (VGAT)-Cre mice (Jackson Laboratory stock 017535, 8–10 weeks). Both male and female VGAT-Cre mice were used in anterograde tracing experiments (8–10 weeks) and in vitro electrophysiological recording (4–6 weeks). Mice were housed at an ambient temperature (22 ± 0.5 °C) with relative humidity of 60% ± 2% and a 12-hour light/dark cycle (lights on at 07:00, illumination intensity approximately 100 lux). Food and water were available ad libitum.
Wild animals	No wild animals in this study.
Reporting on sex	All in vivo results observing sleep–wake behavior by EEG recording were from male mice only in this study.
Field-collected samples	Field-collected samples are not involved in this study.
Ethics oversight	All experimental procedures were approved by the Committee on the Ethics of Animal Experiments of School of Basic Medical Sciences, Fudan University, with license identification number 20210302-105.

Note that full information on the approval of the study protocol must also be provided in the manuscript.
